# Safety of folic acid

**DOI:** 10.1111/nyas.13499

**Published:** 2017-11-20

**Authors:** Martha S. Field, Patrick J. Stover

**Affiliations:** ^1^ Division of Nutritional Sciences Cornell University Ithaca New York

**Keywords:** folic acid, safety, cancer, neural tube defects, folate

## Abstract

There is a large body of literature demonstrating the efficacy of maternal folic acid intake in preventing birth defects, as well as investigations into potential adverse consequences of consuming folic acid above the upper intake level (UL). Recently, two authoritative bodies convened expert panels to assess risks from high intakes of folic acid: the U.S. National Toxicology Program and the UK Scientific Advisory Committee on Nutrition. Overall, the totality of the evidence examined by these panels, as well as studies published since the release of their reports, have not established risks for adverse consequences resulting from existing mandatory folic acid fortification programs that have been implemented in many countries. Current folic acid fortification programs have been shown to support public health in populations, and the exposure levels are informed by and adherent to the precautionary principle. Additional research is needed to assess the health effects of folic acid supplement use when the current upper limit for folic acid is exceeded.

## Introduction

Folic acid intake below the established tolerable upper intake level (UL) of 1000 μg/day for the general population is not associated with any adverse health outcomes. However, public health efforts to reduce the incidence of folic acid–responsive neural tube defects (NTDs) by increasing folic acid intake at levels below the UL have raised questions concerning the safety of elevated folic acid intake. Not all countries require that the food supply be fortified with folic acid for NTD prevention, despite the unequivocal evidence for its beneficial effects on reducing rates of NTDs and potentially other developmental anomalies.^1^ On the other hand, folic acid supplementation specifically targeting women of reproductive age is recommended globally at intake levels of at least 400 μg/day, and up to 5 mg/day for women at high risk.^2^, ^3^ NTDs arise when the neural folds fail to fuse during embryonic development and result in lifelong, debilitating medical conditions. Randomized controlled trials^4^, ^5^ and other trials in the 1980s and 1990s demonstrated that maternal supplementation with multivitamins containing folic acid prevents NTD occurrence^5^ or recurrence.^4^, ^6^, ^7^ In January 2017, the United States Preventative Services Task Force (USPSTF) released a recommendation stating: “The USPSTF concludes with high certainty that the net benefit of daily folic acid supplementation to prevent NTDs in the developing fetus is substantial for women who are planning or capable of pregnancy.”^1^ Other authoritative organizations, including the U.S. Centers for Disease Control and the Institute of Medicine (IOM; renamed the National Academies of Science, Engineering, and Medicine) have made similar recommendations.^8^, ^9^ Neural tube closure occurs in the fourth week after conception. Therefore, the critical window in which to intervene occurs before most women are aware that they are pregnant. Considering that as many as half of all pregnancies in the United States are not planned^10^ and that compliance with public health recommendations for women planning pregnancy to consume folic acid supplements is low,^11^ we propose that universal, mandatory fortification is the most effective strategy for increasing folic acid intakes to prevent NTDs. The Food Fortification Initiative 2017 report lists 86 countries that mandate iron or folic acid fortification of one or more cereal grains.^12^ Mandatory folic acid fortification occurs in 75 countries, but not in 28 European Union member countries.^13^

## Folic acid exposure from fortification

The U.S. Food and Drug Administration's (FDA) regulations on food “standards of identity” specify the ingredients a given food product must contain to market it under a specific name, as is the case for products carrying the “enriched” label as it applies to mandatory fortification programs. Use of the “enriched” label on products requires mandatory fortification with specified micronutrients at specified levels. Voluntary fortification refers to the optional fortification of foods that do not carry a “standard of identify” for enriched products. As described below, folic acid fortification in the United States is considered mandatory, as it applies to flour, grain, and cereal products carrying the “enriched” label. In general, mandatory micronutrient fortification programs result in more uniform exposure of a given micronutrient across individuals in a population compared with voluntary fortification programs, in terms of both coverage and exposure level. In Ireland, a cross‐sectional study that assessed folate and vitamin B_12_ status resulting from a voluntary fortification program and/or supplement use revealed that, although consumption of fortified foods and supplements increased folate status, this increase was not consistent across the population.^14^ Notably, many women of reproductive age (66%) exhibited red blood cell folate levels below the optimal level shown to reduce NTD risk.^14^, ^15^, ^16^

To reduce rates of NTDs in the United States, the FDA mandated that 140 μg of folic acid be added per 100 g of enriched flour, rice, pasta, cornmeal, and other grain products that carry the “enriched” label, with an effective date of January 1, 1998.^17^ Folic acid is a synthetic, fully oxidized form of folate that is used to fortify foods, and this is described in more detail below.^8^ This level of fortification was chosen on the basis of the observation that adding 70 μg folic acid per 100 g product would replace folate lost in processing, whereas adding five times that level (350 μg/100 g) was likely to result in individuals consuming more than the 1000 μg/day UL for folic acid that was established by the IOM, as detailed below.^18^ The addition of 140 μg folic acid per 100 g enriched product was originally estimated to result in an intake of about an additional 100 μg folic acid per day in adults and bring total folic acid intake levels to at least 400 μg/day of folic acid, which is the intake level recommended by the Public Health Service for women of childbearing age,^9^ while keeping most adults below the UL.^17^ Subsequent studies have estimated that fortification in the United States provides 138 μg/day of folic acid intake in addition to folate intake from supplements and other food sources.^19^

## The tolerable upper intake level for folic acid

There is no UL for natural reduced folates found in foods.^8^ The UL for the provitamin folic acid was established to avoid a delayed diagnosis of vitamin B_12_ deficiency, as assessed by hematological indices, and thereby minimize the risk of neurological complications in vitamin B_12_‐deficient individuals. The IOM stated, “The weight of the limited but suggestive evidence that excessive folate intake may precipitate or exacerbate neuropathy in vitamin B_12_‐deficient individuals justifies the selection of this end point as the critical end point for the development of a UL for folate.”^8^ The IOM was careful to note that there was not sufficient evidence to establish a UL on the basis of a no‐observed‐adverse‐effect level (NOAEL) but rather on the basis of a lowest‐observed‐adverse‐effect level (LOAEL).^8^ The LOAEL was set at 5 mg/day on the basis of several case reports and small observational studies showing that, at doses of 5 mg/day folic acid and above, there were more than 100 reported cases (from more than 20 studies) of neurological progression in patients with pernicious anemia, compared with fewer than eight cases in studies administering less than 5 mg/day oral folic acid.^8^ As stated by the IOM, “The LOAEL of 5 mg/day of folate was divided by an uncertainty factor of 5 to obtain the UL for adults of 1 mg/day or 1000 μg/day of folate from supplements or fortified food. A UL of 1000 μg/day is set for all adults rather than just for the elderly because of (1) the devastating and irreversible nature of the neurological consequences of a delayed diagnosis and treatment of a vitamin B_12_ deficiency, (2) data suggesting that pernicious anemia may develop at a younger age in some racial or ethnic groups,^20^ and (3) uncertainty about the occurrence of vitamin B_12_ deficiency in younger age groups.” According to the IOM, “the prevalence of vitamin B_12_ deficiency in females in the childbearing years is very low and the consumption of supplemental folate at or above the UL in this subgroup is unlikely to produce adverse effects,”^8^ although exceptions might include vegetarians, subsets of the population that have low dietary meat intake, and chronic users of proton pump inhibitors. Hematological indices are not commonly used to assess vitamin B_12_ deficiency, as they have been replaced with the use of serum biomarkers;^21^ hence, the basis for the UL for folic acid, which is based on hematological assessment of vitamin B_12_ deficiency, is less meaningful today relative to when it was established nearly 20 years ago.

## The precautionary principle

Some countries have elected not to institute a folic acid fortification program because of concerns of unintended consequences. The “precautionary principle” is perhaps most well known in relation to environmental science and with respect to campaigns designed to limit environmental damage. The original definition states, “when an activity raises threats of harm to human health or the environment, precautionary measures should be taken even if some cause and effect relationships are not fully established scientifically” and was set forth by the Rio Declaration of 1992 and the 1998 Wingspread Statement.^22^ At its core, the precautionary principle aims to anticipate unintended adverse consequences in the implementation of public health interventions and to conduct safety monitoring following an intervention to ensure that the intended benefits are achieved and that unintended adverse consequences are avoided.^22^ There are numerous examples in environmental and/or public health interventions that, while set forth with the best intentions, were subsequently plagued by unintended adverse consequences, such as drilling wells in Bangladesh to guard against diarrheal diseases caused by microbial water contamination that ultimately resulted in arsenic‐contaminated drinking water.^23^ As reviewed below, the experiences of two decades of mandatory folic acid fortification in the United States and Canada, as well as experiences in other countries, have not provided evidence of any adverse effects attributed to mandatory folic acid fortification.^24^

## Unique aspects of folic acid fortification

There are four unique aspects of folic acid fortification of enriched grains and cereal products compared with previous nutrient fortification initiatives, including (1) the health outcome, (2) targeting, (3) fortificant used, and (4) fundamental lack of knowledge of mechanism of action.

First, folic acid fortification was the first nutrient fortification initiative where the primary goal was not to remedy a classical nutrient deficiency in the population on the basis of functional biomarkers of metabolism or a disease attributed solely to a nutrient deficiency in the population, but rather to reduce risk for a clinical condition whose etiology is complex and multifactorial, namely NTDs. The experience with folic acid fortification for NTD prevention has helped transform the paradigm for establishing nutrient requirements. Currently, there are ongoing initiatives to estimate dietary reference intakes using chronic disease end points, as opposed to functional indicators of nutrient deficiency or deficiency‐related disease.^25^ However, consideration of chronic disease prevention and other pathologies with complex etiology to establish nutrient requirements was less common in the 1990s.

Second, the intervention exposed the entire population to folic acid, but was intended for a narrow segment of the population, namely women of reproductive age, especially those who are at increased risk for carrying a folic acid–responsive NTD‐affected pregnancy. NTDs are generally recognized to result from complex gene–nutrient–environment interactions.^26^ Environmental toxins also contribute to risk in limited and specific contexts, such as the contamination of corn‐based products with the mycotoxin fumonisin^27^ or arsenic exposure.^28^ Current knowledge does not allow *a priori* identification of women who are at risk of folic acid–responsive NTDs.

Third, the use of folic acid as the fortificant was controversial, because at high levels of intake it can appear in blood in an unmetabolized form (as described below). Folic acid is a synthetic and chemically stable form of folate and not the natural form of the vitamin found in whole foods. It is highly stable to oxidative degradation and therefore the preferred form of folate used in dietary supplements, ready‐to‐eat breakfast cereals, and fortified food. Folic acid has been present in multivitamin supplements and foods for infants and young children for over 50 years without any evidence of harm when intake levels are below the UL. Physiological forms of folate that function as enzyme cofactors in metabolism include tetrahydrofolates and dihydrofolate. These forms of the vitamin are unstable and often undergo irreversible degradation during food preparation and cooking.^29^ Because of its increased chemical stability and lack of a conjugated polyglutamate peptide compared with natural food folate, folic acid is more bioavailable than natural folate contained in food. Whereas natural food folate is approximately 50% bioavailable, folic acid is 85% bioavailable and hence is ∼1.7 times more bioavailable than food folate. For this reason, folate intake is expressed as dietary folate equivalents, where 1.7 μg of natural food folate equals 1.0 μg of folic acid.^30^

Once transported into a cell, folic acid is converted into the natural reduced forms of folate by the enzyme dihydrofolate reductase, although this reaction is slow and is readily saturated.^31^ Hence, several studies reported the presence of unmetabolized folic acid (UMFA) in serum.^32^ Although the initial evidence indicated that most folic acid was converted into reduced tetrahydrofolate in the epithelial cells of the small intestine, subsequent studies indicated that significant quantities of folic acid are exported into the hepatic portal vein and metabolized into tetrahydrofolates in the liver.^33^ Indeed, folic acid accounts for about 40% of total folate in milk from lactating mothers taking a prenatal supplement containing folic acid.^34^

Folic acid has not been demonstrated to have any meaningful impact on human physiology when intake levels are below the UL. Folic acid has no established biological activity other than binding to folate receptors and transporters for cellular import. Once in the cell, folic acid cannot be converted to polyglutamate forms of folate; it first must be reduced to the tetrahydrofolate to serve as a metabolic cofactor.^29^ This conversion from folate monoglutamate forms that are absorbed to polyglutamate forms is essential for high‐affinity binding of folate cofactors to folate‐dependent enzymes. At the time that folic acid fortification was implemented in the United States, the IOM recognized “…that excessive intake of folate supplements may obscure or mask and potentially delay the diagnosis of vitamin B_12_ deficiency…”^8^ Vitamin B_12_ deficiency can be common in older adults.^35^ In animal models and in humans, vitamin B_12_ deficiency is associated with megaloblastic anemia and neuropathies; while the anemia is reversible, neuropathies can be irreversible. A single study in primates reported that vitamin B_12_‐deficient animals receiving folic acid supplements developed neurological damage earlier than vitamin B_12_‐deficient animals not receiving folic acid.^36^ A biological premise based on knowledge of folate metabolism underlying this finding is lacking, and there is a need to replicate this study in both animal and human cell experimental model systems.

As described above, the UL for folic acid was established to avoid a delayed diagnosis of vitamin B_12_ deficiency owing to high intake of synthetic folic acid in vitamin B_12_‐deficient individuals. It is important to note that the UL was established for folic acid, and not for reduced forms of folate or natural food folate.^8^ It is also important to note that folic acid fortification of foods is only one source of folic acid intake, and recent publications have described the sources of folic acid exposure in the U.S. population. An observational study quantified sources of daily folic acid intake using 24‐h recalls from more than 8000 nonpregnant women in which dietary recall and serum folate levels were available from National Health and Nutrition Examination Survey (NHANES) during 2001–2004. The primary sources of folic acid were foods containing fortified flour, consumption of fortified ready‐to‐eat cereals, and multivitamin supplements. The main finding of this study was that, of the three possible sources of folic acid, supplement use was the primary driver of high serum folate levels in individuals within the highest quintile of serum folate. In fact, folic acid–containing supplements accounted for about 75% of serum folate in this quintile.^37^ In a study that measured both reduced 5‐methyl‐THF (the primary circulating form of folate) and UMFA in serum samples from NHANES 2007–2008, UMFA was detected in serum from nearly all individuals and ranged from 0.3 to 5 nmol/L among individuals; supplement use was shown to increase serum levels of both 5‐methyl‐THF and UMFA.^32^ The UK Scientific Advisory Committee on Nutrition (SACN) recently concluded that data were insufficient to determine the health effects of UMFA.^38^ The presence of UMFA in circulation in humans and rodent models is well characterized;^32^, ^33^, ^39^, ^40^, ^41^ however, the functional and health consequences, if any, of UMFA are not established.

**Figure 1:**
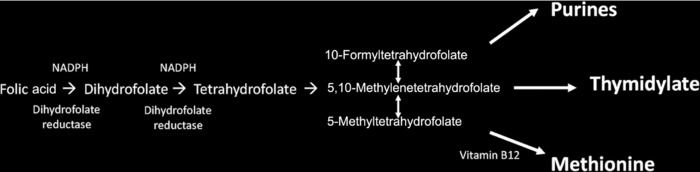
Folate‐mediated one‐carbon metabolism. Dietary folic acid is metabolized to tetrahydrofolate, which is then activated with a one‐carbon unit to form 10‐formyltetrahydrofolate, 5,10‐methylenetetrahydrofolate, and 5‐methyltetrahydrofolate. Each of these folate cofactors supports a biosynthetic pathway for the synthesis of purines and thymidylate and the remethylation of homocysteine to methionine. Synthesis of methionine requires 5‐methyltetrahydrofolate and vitamin B_12_. Folic acid is converted to dihydrofolate and then tetrahydrofolate by dihydrofolate reductase, which is dependent on NADPH.

Fourth, the mechanism whereby folic acid prevents NTDs is unknown, limiting the ability to predict unintended clinical consequences associated with fortification on the basis of knowledge of the fundamental folate biochemistry and pathophysiology. The folate‐dependent metabolic pathway that causes risk for NTDs has not been established. Folates function in the cell as cofactors that carry chemically activated one‐carbon units for a network of biosynthetic reactions known as one‐carbon metabolism, which includes the *de novo* synthesis of purines (adenine and guanine), *de novo* thymidylate (dTMP) biosynthesis, and the remethylation of homocysteine to methionine (Fig. 1).^42^ There are established biomarkers of impaired folate metabolism for each of the pathways within the network.^43^ Impaired homocysteine remethylation is associated with increased plasma homocysteine concentrations and DNA hypomethylation, whereas impaired dTMP and purine synthesis results in decreased rates of DNA replication, which impairs cell division, and increased uracil misincorporation into DNA.^42^ Because folate functions in such highly interconnected metabolic pathways that directly affect genome stability and genome expression, defining causal relationships linking folate‐associated pathologies to impairment in any one pathway within the metabolic network is challenging.^44^ Although folic acid supplementation was shown to be efficacious in preventing NTDs in controlled trials, a deeper understanding of the fundamental biological mechanism linking folic acid intake to NTD prevention is needed to better target the intervention to those at risk and potentially determine the role of folate in health and disease more broadly.

## Safety of folic acid

There is a large body of literature demonstrating the efficacy of maternal folic acid intake in preventing birth defects, as well as investigations into potential adverse consequences of consuming folic acid above the UL. Recently, two separate authoritative bodies convened expert panels to assess the risks from high intakes of folic acid. In May 2015, the U.S. National Toxicology Program (NTP) and the Office of Dietary Supplements convened an expert panel and performed systematic reviews of existing literature regarding the safe use of high folic acid intakes and was charged with developing future research priorities.^45^ The NTP panel was tasked with (1) identifying the areas of consistency and uncertainty in current science, (2) identifying research needs given the state of the science, and (3) proposing approaches to address knowledge gaps.^45^ This group was divided into several subpanels, with each subpanel addressing an area of concern, including cancer, cognition (especially as related to interactions with vitamin B_12_ deficiency), hypersensitivity‐related outcomes, and thyroid‐ and diabetes‐related disorders. These areas of concern were based on previous studies reporting adverse effects in these health effect categories with intakes of greater than 400 μg/day folic acid, serum folates greater than 10 nM/L, or red blood cell folate greater than 340 nM/L.^46^ The overall conclusion of the NTP report was that, for the outcomes considered, there was no conclusive evidence for adverse effects because of folic acid, but, in each case, a research agenda was proposed to address current knowledge gaps, as described below.

The UK SACN comprises appointed scientific experts who advise Public Health England and other UK governmental organizations on nutrition. A SACN panel was convened in April 2016 to update their previous recommendations in favor of mandatory folic acid fortification that were published in 2006 and 2009 and to review the literature on potential adverse effects of folic acid intake. A draft report submitted to public consultation in early 2017^38^ focused on putative adverse effects of folic acid fortification that included masking/exacerbation of low vitamin B_12_ status, cognitive decline in the elderly, cancer, and the appearance of UMFA in the systemic circulation. This group was tasked with conducting a review of new evidence, relying heavily on systematic reviews and meta‐analyses for all outcomes except UMFA in circulation, as there were fewer studies identified for this outcome.^38^

Additionally, the SACN summarized the results of five other evaluations conducted after their 2006 review, one of which was the NTP report.^46^ The Food Safety Authority of Ireland produced an updated report on folic acid and prevention of NTDs in 2016;^47^ the FDA summarized outcomes and conclusions for their Memorandum for Food Additive petition for folic acid in corn masa flour in 2016;^48^ the Norwegian Scientific Committee for Food Safety (VKM) conducted a risk assessment of folic acid in food supplements in 2015;^49^ and the European Food Safety Authority evaluated outcomes and provided scientific opinion on dietary reference values for folate.^50^

The following section provides an overview of each major putative adverse topic and then focuses on the NTP and SACN expert reviews summarized below by disease outcome, as well as relevant recent literature that was not included in the SCAN or NTP reports. Later in 2017, the SACN will offer a recommendation on whether its previous recommendations regarding mandatory folic acid fortification still applies for the United Kingdom.

It is worth noting that both the NTP and SACN reports focus on population and clinical data from high‐income settings, although the application of these findings is expected to be widely applicable to low‐ and middle‐income contexts.

### Cancer

A biological premise underpinning the role of folic acid intake and adverse cancer outcomes has been published.^51^ The proposed effects of folic acid on cancer risk are not related to folic acid per se, but rather relate to its effect in elevating whole‐body folate status. It is proposed that folate deficiency increases the initiation of carcinogenesis by causing genome instability as well as alteration of methylation patterns in the genome, leading to altered expression of oncogenes and tumor suppressor genes. Elevated folate status is postulated to promote carcinogenesis and tumor growth once the transformation process has been initiated by increasing synthesis of nucleotides and other metabolites required for replication and expression of the genome.^51^, ^52^ Evidence and gaps related to this mechanism are summarized below.

There is strong evidence that low folate status promotes cancer, especially colorectal cancer.^51^ As noted in the NTP report, there is both preclinical data from rodent models and human observational and clinical evidence to indicate that inadequate folate intake from all sources is associated with increased cancer risk.^46^ Because folate cofactors are required for nucleotide biosynthesis and for generating activated methyl groups that modify chromatin and affect gene expression, low‐folate status has been demonstrated to decrease rates of nucleotide biosynthesis and impair chromatin methylation. Hence, low‐folate status promotes genome instability and DNA mutation rates and alters the methylation patterns in the genome that affect the expression of tumor suppressor genes.^52^, ^53^ There is strong consensus in the scientific community that folate deficiency increases risk for certain cancers.

Elevated folate status has been proposed to promote tumorigenesis from preexisting foci of neoplasia and accelerate the growth of established tumors.^52^ The biological premise underlying this hypothesis relates to the role of folate in the synthesis of nucleotides for DNA synthesis in rapidly dividing cells and the use of antifolate chemotherapeutics (methotrexate, pemetrexed, and 5‐flurouracil, among others) that target and inhibit folate‐dependent enzymes and inhibit cell proliferation. Antifolates have been used successfully in the treatment of many types of cancer, and some postulate that, by increasing nucleotide synthesis for cellular proliferation, folate from all sources could accelerate tumor growth.^51^ However, evidence demonstrating a dose–response relationship between folate status and/or folate/folic acid intake within the normal human exposure ranges and increased rates of tumor growth *in vivo* are lacking, and it has not been established whether folate availability is rate limiting in tumor growth in individuals with adequate folate status.

The totality of the evidence from randomized controlled trials does not support folic acid supplementation or fortification increasing cancer risk. A recent meta‐analysis of randomized controlled trials that included over 50,000 individuals examined the relationship between folic acid supplementation (with folic acid doses approximately an order of magnitude greater than exposure levels from folic acid fortification) and cancer incidence from trials conducted before 2011. The analysis included site‐specific cancer rates.^54^ The results of the meta‐analysis indicated that “there was no significant effect of folic acid supplementation (with a median dose of 2.0 mg/day folic acid) on the incidence of cancer of the large intestine, prostate, lung, breast, or any other specific site.”^54^ The study also indicated that cancer mortality rates have continued to decline since the initiation of mandatory folic acid fortification in the United States. Studies since the NTP review support this finding and include a meta‐analysis of folic acid supplementation and colorectal cancer risk.^55^

The NTP panel noted several gaps in current knowledge linking high folic acid intakes to cancer outcomes from animal model studies and human studies and highlighted the importance of understanding “whether existing evidence from clinical trials for increased risk is driven by effects in specific subgroups,” as well as identifying other interacting modifiers of the effect of folic acid, such as age, preexisting neoplasia, and/or genetics (i.e., identify predisposed subgroups).^46^ In these cases, the primary concern relates to intake of folic acid from dietary supplements.

In 2016, the SACN noted that many studies investigating the effects of folic acid intakes on cancer risk may not have been adequately powered to detect changes in cancer incidence, as many studies were not designed specifically for this outcome. Similarly, the committee noted that many of the studies may not have been of sufficient duration to detect an increase in cancer incidence, as cancer is known to develop over many years.^38^ The committee concluded that data linking prostate cancer risk to folic acid and/or folate intake were inconclusive, but noted that two observational studies measuring serum folate levels demonstrated a positive correlation between serum folate levels and prostate cancer risk.^38^ The SACN committee also concluded that there were insufficient data to show that folic acid increases breast cancer risk. Results from both controlled trials and observational studies related to colorectal cancer risk were determined to be highly heterogeneous. Although meta‐analyses have failed to detect an effect of folic acid on colorectal cancer risk, the SACN committee again noted that statistical power may have been inadequate to detect this risk. Others have proposed that meta‐analyses may not detect cancer‐promoting effects of high folic acid intake that may occur in select circumstances, including in individuals who may be sensitized to elevated folic acid intake.^56^

As stated in the NTP report, data reporting on the relationship between folic acid and colon cancer in rodent models have been inconsistent^46^ and highlight the need to develop a research agenda using well‐designed animal model systems. This conclusion was echoed in a 2012 systematic review of the evidence of the effects of dietary folate/folic acid and methionine in the *Apc* ^Min^ *^/+^* mouse model of spontaneous intestinal tumor formation.^57^ The review found that some studies indicate an inhibitory effect of dietary folic acid on tumor development, while other studies indicate increased tumor growth with increased dietary folic acid. Another conclusion of Teh *et al*. was that timing and dose of intervention varied markedly among studies in the *Apc* ^Min^ *^/+^* mouse model, some studies used folic acid doses 10‐fold higher than what is considered adequate for mouse chow.^57^ In general, dietary studies using animals should model folic acid intakes consistent with human exposures. In many studies, comparisons are made between severely folate‐deficient mice and mice exposed to high levels of folic acid intake. The NTP report highlighted the importance of choosing appropriate animal models to study the effects of folic acid on cancer risk, understanding the effects of the timing and exposure of the intervention on cancer outcomes, comparing the effects of folic acid and reduced folates, and understanding dose–response relationships.^45^ This includes comparing outcomes from mice with adequate folate status to those with elevated status at levels consistent with human exposures. Also important is limiting the exposure to folic acid to intake levels consistent with what is observed in human populations.

### Cognitive outcomes

The hypothesis that folic acid intake causes or accelerates cognitive impairment resulting from vitamin B_12_ deficiency is founded on case studies and observational studies. Folate and vitamin B_12_ interact in folate‐mediated one‐carbon metabolism. The two enzymatic cofactors converge in the homocysteine remethylation pathway, where they are both required by the enzyme methionine synthase for the remethylation of homocysteine to methionine. Hence, deficiency of either vitamin leads to elevated serum homocysteine.^43^ Vitamin B_12_ deficiency also induces a secondary, functional folate deficiency, known as a “5‐methyltetrahydrofolate trap.” Because methionine synthase is the only enzyme that metabolizes 5‐methyltetrahydrofolate, vitamin B_12_ deficiency results in its accumulation, thus “starving” other folate‐dependent metabolic pathways for folate cofactors, including nucleotide biosynthesis. Hence, one of the initial clinical presentations of vitamin B_12_ deficiency is megaloblastic anemia owing to inhibition of DNA synthesis. Dietary folic acid can rescue the anemia by providing new pools of cofactors for DNA synthesis that initially escape the methyl trap. However, elevated folic acid intake is not known to rescue neurological pathology resulting from vitamin B_12_ deficiency. There are no proposed biological premises or suggested mechanisms whereby elevated folic acid intake exacerbates vitamin B_12_ deficiency at the level of metabolism, cellular physiology, or human pathogenesis.^58^

Concerns for adverse effects of high folic acid intakes on cognition in vitamin B_12_ deficiency arose initially from case reports.^59^ An observational study of data collected in the NHANES found that “In seniors with low vitamin B_12_ status, high serum folate was associated with anemia and cognitive impairment. When vitamin B_12_ status was normal, however, high serum folate was associated with protection against cognitive impairment.”^60^ Other studies have observed the highest rates of cognitive decline with low vitamin B_12_ status in the presence of high plasma folate or use of folic acid supplements.^61^ Similarly, metabolic markers of vitamin B_12_ deficiency are more pronounced at higher levels of plasma folate.^62^ However, this association is increasingly being interpreted as resulting from the inclusion of individuals who consume nutritional supplements who readily absorb folate but poorly absorb vitamin B_12;_ with vitamin B_12_ malabsorption driving the association.^61^

To date, there are no results from controlled trials testing the association of elevated folate status with low vitamin B_12_ status on accelerating cognitive decline, nor would they be ethical to perform in humans; the few studies supporting this finding are observational.^46^ Furthermore, there is significant heterogeneity among these observational studies with respect to vitamin status cut points and cognitive outcomes assessed, and a general lack of key neurological outcomes.^46^ Observational studies in nutrition generally have a poor track record when tested in follow‐up randomized trials, with one report showing that none of the 52 correlations from 12 observational studies were validated in subsequent randomized clinical trials.^63^ There are no data from animal models supporting the human observational data.

A recent study published after the NTP report^46^ described a secondary analysis of data from a clinical trial among asymptomatic elderly Chileans with low vitamin B_12_ status that tested the efficacy of a single intramuscular injection of a vitamin mixture containing vitamin B_12_, vitamin B_6_, and vitamin B_1_ on neurophysiological function. This secondary analysis examined the association between subjects’ baseline folate status and their response at a single time point 4 months after the multivitamin treatment using a newly proposed combined status and functional vitamin B_12_ indicator (cB‐12). The cB‐12 indicator is a computed variable that combines plasma homocysteine concentration and serum concentrations of methylmalonic acid, holotranscobalamin, and vitamin B_12_. The authors reported weaker response of cB‐12 to the multivitamin treatment among subjects with serum folate levels above the median (33.9 nmol/L).^64^ The authors concluded that improvement in vitamin B_12_ status in deficient individuals after intramuscular B_12_ supplementation is attenuated by high folate status. Interestingly, baseline folate status was not associated with differences in response to the multivitamin treatment as related to nerve function, a primary outcome of the registered trial.^64^ It is important to remember that the interaction between folate status and response to vitamin B_12_ treatment was neither a primary nor secondary outcome of the trial as registered. The strength of the evidence from this secondary data analysis is weak, which limits any conclusions drawn. The cB‐12 indicator is a relatively new concept that is not used clinically and has had limited evaluation by the research community. Evaluated individually, none of the four biochemical measures used to compute the cB‐12 had an interaction with folate status. The finding was only evaluated at 4 months after treatment and does not correlate with any clearly defined clinical outcome. Given the close biochemical relationship between folate and vitamin B_12_, it is biologically plausible that, among individuals with low vitamin B_12_ status, those with different underlying folate status could have different short‐term responses to injections of vitamin B_12_. It is well established in the literature that elevated intake of folate or folic acid can functionally compensate for vitamin B_12_ deficiency, as first reported for the masking of vitamin B_12_ deficiency–related megaloblastic anemia by folic acid.^58^ Hence, vitamin B_12_‐deficient individuals with elevated folate status would be expected to exhibit less improvement in functional indicators as a result of vitamin B_12_ therapy than those with lower folate status because of the functional compensation afforded by folate in vitamin B_12_ deficiency. A well‐designed clinical trial among individuals with low vitamin B_12_ status to evaluate the association between clearly defined clinical outcomes and appropriate measures of folate status would be needed to validate these purported findings.^64^

The SACN committee concluded that folic acid did not have a significant effect on cognitive decline in intervention trials, though it was also noted that studies were generally of short duration and neither focused on nor powered for assessment of effects in individuals with low vitamin B_12_ status. The SACN also stated that observational studies showed either no risk or protection from cognitive decline with higher folate status among individuals with normal vitamin B_12_ status.^38^

The SACN addressed concerns that folic acid fortification may mask signs of vitamin B_12_ deficiency. The committee cited evidence indicating that the prevalence of vitamin B_12_ deficiency as defined by serum vitamin B_12_ less than 148 pmol/L was not affected by mandatory folic acid fortification in the United States, although it was also noted that the biochemical measures used to assess vitamin B_12_ deficiency are of limited reliability and that there is variation in cut off values used to define deficiency.^38^

### Diabetes‐related disorders and thyroid disease

Concern over adverse effects of high folic acid intakes on diabetes‐related disorders and/or thyroid disease arose from conflicting findings from two observational studies^65^, ^66^ and the biological premise that maternal folate (or one‐carbon donor sources) can effect embryonic DNA and/or chromatin methylation and program gene expression in offspring with effects that persist into adulthood.^67^, ^68^, ^69^, ^70^ A longitudinal study in India examined the effects of maternal folic acid and iron supplementation during pregnancy (100 tablets of 500 μg folic acid and 60 mg iron from 18 weeks gestation) on insulin resistance, as assessed by the homeostatic model assessment of insulin resistance in 6‐year‐old children. The pregnant women were from six villages in rural India and were primarily low income and vegetarian and exhibited low vitamin B_12_ status and adequate folate status.^65^ The offspring of mothers with high serum folate and low serum vitamin B_12_ levels were the most insulin resistant, leading the authors to conclude that “Low maternal vitamin B_12_ and high folate status may contribute to the epidemic of adiposity and type 2 diabetes in India.”^65^ However, a related randomized controlled trial conducted in rural Nepal in which pregnant mothers were supplemented with a series of micronutrients, including vitamin A (1 mg retinol equivalents/day), folic acid (400 μg/day), iron (60 mg/day), and zinc (30 mg/day), did not confirm this association.^66^ In fact, children of mothers supplemented daily with 400 μg folic acid and vitamin A exhibited significantly lower risk of metabolic syndrome than children of mothers supplemented with only vitamin A (the control group).^66^

The NTP panel concluded that future work should follow up on the observational findings as related to prenatal exposures and that, on the basis of this very limited data, there was no consistent evidence that high intakes of folic acid and/or high folate status influences diabetes risk or glucose metabolism.^45^, ^46^

### Hypersensitivity‐related outcomes

As noted in the NTP report, observational studies reporting hypersensitivity‐related outcomes, such as childhood asthma and allergy, have emerged over the past 20 years.^46^ Concern for adverse effects of maternal folic acid intake are again related to the ability of folate and/or methyl donors to program fetal gene expression.^67^, ^68^, ^69^, ^70^ The NTP panel concluded that, with respect to sensitization to asthma, data on the effects of high folic acid intakes were limited.^45^ The panel also emphasized the need to understand whether folic acid functions in biological pathways leading to asthma sensitization and to perform rigorous controlled human studies in pregnant women and in children to better assess the mechanisms and risk.^45^ In terms of risk for hypersensitivity outcomes, such as eczema and respiratory infections, the panel concluded that this is not a priority research area owing to lack of available data.^45^

### Cardiovascular disease, twinning, autism, mortality, immunological outcomes, other neurological outcomes, and other reproductive outcomes

There is scant literature that has proposed that these disease outcomes are associated with high folic acid intake. The NTP systematic review process did not find sufficient evidence to warrant consideration of this literature by the panel owing to lack of sufficient evidence of adverse effects.^46^ Furthermore, connecting folic acid to these outcomes lacks a strong biological and mechanistic premise. In some cases, folate intakes from all sources at or above the recommended dietary allowance and/or maintenance of adequate folate status have been shown to be protective for these and related outcomes.^71^, ^72^, ^73^, ^74^, ^75^, ^76^

## Summary and conclusions

Here, the evidence for the safety of folic acid fortification was reviewed, primarily on the basis of very thorough assessments conducted by the U.S. NTP in 2015 and the UK SACN in 2016. Neither the SACN nor the NTP reports identified conclusive evidence for adverse effects of folic acid. The NTP report emphasizes the uncertainty that exists in the present literature, proposes a research agenda, and emphasizes that “additional work is critical to fully evaluating the known public health benefits of folic acid, as well as the potential—but still unevaluated—risks that may exist.”^45^, ^46^ The SACN 2016 draft report reviewed literature published since their 2006 and 2009 recommendations for mandatory fortification of flour with folic acid^38^ and, after public consultation of the draft, is now assessing whether its previous recommendations still stand.

Overall, the totality of the evidence of the literature fully supports the benefits of mandatory folic acid fortification in NTD prevention. Furthermore, there are no established risks for adverse consequences resulting from existing mandatory folic acid fortification programs that have been implemented in many countries. Current folic acid fortification programs have been shown to support public health in populations, and the exposure levels are informed by and adherent to the precautionary principle. Additional research is needed to assess the health effects of folic acid supplement use when the current UL for folic acid is exceeded.

## Competing interests

The authors declare no competing interests.

## References

[1] Bibbins‐Domingo, K. , D.C. Grossman , S.J. Curry , *et al* 2017 Folic acid supplementation for the prevention of neural tube defects: US Preventive Services Task Force Recommendation Statement. JAMA 317: 183–189. 28097362 10.1001/jama.2016.19438

[2] Gomes, S. , C. Lopes & E. Pinto . 2016 Folate and folic acid in the periconceptional period: recommendations from official health organizations in thirty‐six countries worldwide and WHO. Public Health Nutr. 19: 176–189. 25877429 10.1017/S1368980015000555 PMC10270901

[3] Moussa, H.N. , S. Hosseini Nasab , Z.A. Haidar , *et al* 2016 Folic acid supplementation: what is new? Fetal, obstetric, long‐term benefits and risks. Future Sci. OA 2: FSO116. 28031963 10.4155/fsoa-2015-0015 PMC5137972

[4] MRC Vitamin Study Research Group . 1991 Prevention of neural tube defects: results of the Medical Research Council Vitamin Study. Lancet. 338: 131–137. 1677062

[5] Czeizel, A. & I. Dudas . 1992 Prevention of the first occurrence of neural tube defects by periconceptional vitamin supplementation. N. Engl. J. Med. 327: 1832–1835. 1307234 10.1056/NEJM199212243272602

[6] Smithells, R.W. , S. Sheppard , J. Wild & C.J. Schorah . 1989 Prevention of neural tube defect recurrences in Yorkshire: final report. Lancet 2: 498–499. 10.1016/s0140-6736(89)92103-x 2570200

[7] Nevin, N.C. & M.J. Seller . 1990 Prevention of neural‐tube‐defect recurrences. Lancet 335: 178–179. 10.1016/0140-6736(90)90060-i 1967478

[8] Standing Committee on the Scientific Evaluation of Dietary Reference Intakes . 1998 Dietary reference intakes for thiamin, riboflavin, niacin, vitamin B6, folate, vitamin B12, pantothenic acid, biotin, and choline. Institute of Medicine (US) Food and Nutrition Board, Washington, DC. 23193625

[9] Centers for Disease Control . 1992 Recommendations for the use of folic acid to reduce the number of cases of spina bifida and other neural tube defects. *MMWR Recomm. Rep* 41(RR‐14): 1–7. 1522835

[10] Finer, L.B. & S.K. Henshaw . 2006 Disparities in rates of unintended pregnancy in the United States, 1994 and 2001. Perspect. Sex. Reprod. Health 38: 90–96. 16772190 10.1363/psrh.38.090.06

[11] Crozier, S.R. , S.M. Robinson , S.E. Borland , *et al* 2009 Do women change their health behaviours in pregnancy? Findings from the Southampton Women's Survey. Paediatr. Perinat. Epidemiol. 23: 446–453. 19689495 10.1111/j.1365-3016.2009.01036.x PMC3091015

[12] Food Fortification Initiative . 2016 Say Hello to a Fortified Future, 2016 in Review. Atlanta, GA: Food Fortification Initiative.

[13] Searby, L. 2016 Folic acid fortification: the current global state of play. Accessed August 30, 2017. http://www.nutraingredients.com/Regulation-Policy/Folic-acid-fortification-The-current-global-state-of-play.

[14] Hopkins, S.M. , M.J. Gibney , A.P. Nugent , *et al* 2015 Impact of voluntary fortification and supplement use on dietary intakes and biomarker status of folate and vitamin B‐12 in Irish adults. Am. J. Clin. Nutr. 101: 1163–1172. 25877491 10.3945/ajcn.115.107151

[15] Daly, L.E. , P.N. Kirke , A. Molloy , *et al* 1995 Folate levels and neural tube defects. Implications for prevention. JAMA 274: 1698–1702. 7474275 10.1001/jama.1995.03530210052030

[16] Crider, K.S. , O. Devine , L. Hao , *et al* 2014 Population red blood cell folate concentrations for prevention of neural tube defects: Bayesian model. BMJ 349: g4554. 25073783 10.1136/bmj.g4554 PMC4115151

[17] Food and Drug Administration‐HHS . 1993 Food standards: amendment of the standards of identity for enriched grain products to require addition of folic acid. Fed. Reg. 58: 53305–53312.

[18] Bailey, L.B. 2004 Folate and vitamin B12 recommended intakes and status in the United States. Nutr. Rev. 62: S14–S20; discussion S21. 15298443 10.1111/j.1753-4887.2004.tb00065.x

[19] Yang, Q. , M.E. Cogswell , H.C. Hamner , *et al* 2010 Folic acid source, usual intake, and folate and vitamin B‐12 status in US adults: National Health and Nutrition Examination Survey (NHANES) 2003–2006. Am. J. Clin. Nutr. 91: 64–72. 19828716 10.3945/ajcn.2009.28401

[20] Carmel, R. & C.S. Johnson . 1978 Racial patterns in pernicious anemia. Early age at onset and increased frequency of intrinsic‐factor antibody in black women. N. Engl. J. Med. 298: 647–650. 628388 10.1056/NEJM197803232981203

[21] Hannibal, L. , V. Lysne , A.L. Bjorke‐Monsen , *et al* 2016 Biomarkers and algorithms for the diagnosis of vitamin B12 deficiency. Front. Mol. Biosci. 3: 27. 27446930 10.3389/fmolb.2016.00027 PMC4921487

[22] Kriebel, D. & J. Tickner . 2001 Reenergizing public health through precaution. Am. J. Public Health 91: 1351–1355. 11527753 10.2105/ajph.91.9.1351 PMC1446776

[23] Goldstein, B.D. 2001 The precautionary principle also applies to public health actions. Am. J. Public Health 91: 1358–1361. 11527755 10.2105/ajph.91.9.1358 PMC1446778

[24] World Health Organization . 2015 Optimal serum and red blood cell folate concentrations in women of reproductive age for prevention of neural tube defects. Geneva: World Health Organization. 25996016

[25] Yetley, E.A. , A.J. MacFarlane , L.S. Greene‐Finestone , *et al* 2017 Options for basing Dietary Reference Intakes (DRIs) on chronic disease endpoints: report from a joint US‐/Canadian‐sponsored working group. Am. J. Clin. Nutr. 105: 249S–285S. 27927637 10.3945/ajcn.116.139097 PMC5183726

[26] Detrait, E.R. , T.M. George , H.C. Etchevers , *et al* 2005 Human neural tube defects: developmental biology, epidemiology, and genetics. Neurotoxicol. Teratol. 27: 515–524. 15939212 10.1016/j.ntt.2004.12.007 PMC2727639

[27] Gelineau‐van Waes, J. , M.A. Rainey , J.R. Maddox , *et al* 2012 Increased sphingoid base‐1‐phosphates and failure of neural tube closure after exposure to fumonisin or FTY720. Birth Defects Res. A Clin. Mol. Teratol. 94: 790–803. 22991331 10.1002/bdra.23074

[28] Mazumdar, M. , M.O. Ibne Hasan , R. Hamid , *et al* 2015 Arsenic is associated with reduced effect of folic acid in myelomeningocele prevention: a case control study in Bangladesh. Environ. Health 14: 34. 25885259 10.1186/s12940-015-0020-0 PMC4404044

[29] Suh, J.R. , A.K. Herbig & P.J. Stover . 2001 New perspectives on folate catabolism. Annu. Rev. Nutr. 21: 255–282. 11375437 10.1146/annurev.nutr.21.1.255

[30] Suitor, C.W. & L.B. Bailey . 2000 Dietary folate equivalents: interpretation and application. J. Am. Diet. Assoc. 100: 88–94. 10646010 10.1016/S0002-8223(00)00027-4

[31] Bailey, S.W. & J.E. Ayling . 2009 The extremely slow and variable activity of dihydrofolate reductase in human liver and its implications for high folic acid intake. Proc. Natl. Acad. Sci. USA 106: 15424–15429. 19706381 10.1073/pnas.0902072106 PMC2730961

[32] Pfeiffer, C.M. , M.R. Sternberg , Z. Fazili , *et al* 2015 Unmetabolized folic acid is detected in nearly all serum samples from US children, adolescents, and adults. J. Nutr. 145: 520–531. 25733468 10.3945/jn.114.201210 PMC4336532

[33] Wright, A.J. , P.M. Finglas , J.R. Dainty , *et al* 2005 Differential kinetic behavior and distribution for pteroylglutamic acid and reduced folates: a revised hypothesis of the primary site of PteGlu metabolism in humans. J. Nutr. 135: 619–623. 15735104 10.1093/jn/135.3.619

[34] West, A.A. , J. Yan , C.A. Perry , *et al* 2012 Folate‐status response to a controlled folate intake in nonpregnant, pregnant, and lactating women. Am. J. Clin. Nutr. 96: 789–800. 22932279 10.3945/ajcn.112.037523

[35] Carmel, R. 2008 Nutritional anemias and the elderly. Semin. Hematol. 45: 225–234. 18809092 10.1053/j.seminhematol.2008.07.009

[36] Agamanolis, D.P. , E.M. Chester , M. Victor , *et al* 1976 Neuropathology of experimental vitamin B12 deficiency in monkeys. Neurology 26: 905–914. 822371 10.1212/wnl.26.10.905

[37] Yeung, L. , Q. Yang & R.J. Berry . 2008 Contributions of total daily intake of folic acid to serum folate concentrations. JAMA 300: 2486–2487. 19050191 10.1001/jama.2008.742

[38] Scientific Advisory Committee on Nutrition . 2017 Draft–update on folic acid. GOV.UK.

[39] Troen, A.M. , B. Mitchell , B. Sorensen , *et al* 2006 Unmetabolized folic acid in plasma is associated with reduced natural killer cell cytotoxicity among postmenopausal women. J. Nutr. 136: 189–194. 16365081 10.1093/jn/136.1.189

[40] Kelly, P. , J. McPartlin , M. Goggins , *et al* 1997 Unmetabolized folic acid in serum: acute studies in subjects consuming fortified food and supplements. Am. J. Clin. Nutr. 65: 1790–1795. 9174474 10.1093/ajcn/65.6.1790

[41] Pfeiffer, C.M. , M.R. Sternberg , Z. Fazili , *et al* 2015 Folate status and concentrations of serum folate forms in the US population: National Health and Nutrition Examination Survey 2011–2012. Br. J. Nutr. 113: 1965–1977. 25917925 10.1017/S0007114515001142 PMC4804191

[42] Fox, J.T. & P.J. Stover . 2008 Folate‐mediated one‐carbon metabolism. Vitam. Horm. 79: 1–44. 18804690 10.1016/S0083-6729(08)00401-9

[43] Yetley, E.A. , C.M. Pfeiffer , K.W. Phinney , *et al* 2011 Biomarkers of folate status in NHANES: a roundtable summary. Am. J. Clin. Nutr. 94: 303S–312S. 21593502 10.3945/ajcn.111.013011 PMC3127517

[44] Misselbeck, K. , L. Marchetti , M.S. Field , *et al* 2017 A hybrid stochastic model of folate‐mediated one‐carbon metabolism: effect of the common C677T MTHFR variant on *de novo* thymidylate biosynthesis. Sci. Rep. 7: 797. 28400561 10.1038/s41598-017-00854-w PMC5429759

[45] Boyles, A.L. , E.A. Yetley , K.A. Thayer & P.M. Coates . 2016 Safe use of high intakes of folic acid: research challenges and paths forward. Nutr. Rev. 74: 469–474. 27272334 10.1093/nutrit/nuw015 PMC5009460

[46] National Toxicology Program . 2015 NTP monograph: identifying research needs for assessing safe use of high intakes of folic acid. Research Triangle Park, NC: National Toxicology Program. Accessed September 28, 2017. https://ntp.niehs.nih.gov/ntp/ohat/folicacid/final_monograph_508.pdf.

[47] Food Safety Authority of Ireland . 2016 Update report on folic acid and the prevention of birth defects in Ireland. Dublin: Food Safety Authority of Ireland.

[48] Food and Drug Administration HHS . 2016 Food additives permitted for direct addition to food for human consumption; folic acid. Final rule. Fed. Reg. 81: 22176–22183. 27101640

[49] Norwegian Scientific Committee for Food Safety . 2015 Risk assessment of folic acid and food supplements; opinion of the panel on nutrition, dietetic products, novel food and allergy of the Norwegian Scientific Committee for Food Safety (VKM). Oslo: Norwegian Scientific Committee for Food Safety.

[50] European Food Safety Authority . 2014 Update report on folic acid and the prevention of birth defects in Ireland. Parma: European Food Safety Authority.

[51] Smith, A.D. , Y.I. Kim & H. Refsum . 2008 Is folic acid good for everyone? Am. J. Clin. Nutr. 87: 517–533. 18326588 10.1093/ajcn/87.3.517

[52] Mason, J.B. 2009 Folate, cancer risk, and the Greek god, Proteus: a tale of two chameleons. Nutr. Rev. 67: 206–212. 10.1111/j.1753-4887.2009.00190.x PMC2763118 19335714

[53] Kim, Y.I. 2006 Folate: a magic bullet or a double edged sword for colorectal cancer prevention? Gut 55: 1387–1389. 16966698 10.1136/gut.2006.095463 PMC1856406

[54] Vollset, S.E. , R. Clarke , S. Lewington , *et al* 2013 Effects of folic acid supplementation on overall and site‐specific cancer incidence during the randomised trials: meta‐analyses of data on 50,000 individuals. Lancet 381: 1029–1036. 23352552 10.1016/S0140-6736(12)62001-7 PMC3836669

[55] Qin, T. , M. Du , H. Du , *et al* 2015 Folic acid supplements and colorectal cancer risk: meta‐analysis of randomized controlled trials. Sci. Rep. 5: 12044. 26131763 10.1038/srep12044 PMC4487230

[56] Mason, J.B. 2011 Folate consumption and cancer risk: a confirmation and some reassurance, but we're not out of the woods quite yet. Am. J. Clin. Nutr. 94: 965–966. 21900462 10.3945/ajcn.111.023796 PMC3173030

[57] Teh, A.H. , E. Symonds , C. Bull , *et al* 2012 The influence of folate and methionine on intestinal tumour development in the *Apc* ^Min^ *^/+^* mouse model. Mut. Res. [Epub ahead of print.] 10.1016/j.mrrev.2012.05.001 22627043

[58] Palmer, A.M. , E. Kamynina , M.S. Field & P.J. Stover . 2017 Folate rescues vitamin B12 depletion‐induced inhibition of nuclear thymidylate biosynthesis and genome instability. Proc. Natl. Acad. Sci. USA. 114: E4095–E4102. 28461497 10.1073/pnas.1619582114 PMC5441772

[59] Lindenbaum, J. , E.B. Healton , D.G. Savage , *et al* 1995 Neuropsychiatric disorders caused by cobalamin deficiency in the absence of anemia or macrocytosis. 1988. Nutrition 11: 181; discussion 180, 182. 7647490

[60] Morris, M.S. , P.F. Jacques , I.H. Rosenberg & J. Selhub . 2007 Folate and vitamin B‐12 status in relation to anemia, macrocytosis, and cognitive impairment in older Americans in the age of folic acid fortification. Am. J. Clin. Nutr. 85: 193–200. 17209196 10.1093/ajcn/85.1.193 PMC1828842

[61] Morris, M.S. , J. Selhub & P.F. Jacques . 2012 Vitamin B‐12 and folate status in relation to decline in scores on the mini‐mental state examination in the Framingham Heart Study. J. Am. Geriatr. Soc. 60: 1457–1464. 22788704 10.1111/j.1532-5415.2012.04076.x PMC3419282

[62] Miller, J.W. , M.G. Garrod , L.H. Allen , *et al* 2009 Metabolic evidence of vitamin B‐12 deficiency, including high homocysteine and methylmalonic acid and low holotranscobalamin, is more pronounced in older adults with elevated plasma folate. Am. J. Clin. Nutr. 90: 1586–1592. 19726595 10.3945/ajcn.2009.27514 PMC2777470

[63] Young, S.S. & A. Karr . 2011 Deming, data and observational studies. Significance 8:116–120.

[64] Brito, A. , R. Verdugo , E. Hertrampf , *et al* 2016 Vitamin B‐12 treatment of asymptomatic, deficient, elderly Chileans improves conductivity in myelinated peripheral nerves, but high serum folate impairs vitamin B‐12 status response assessed by the combined indicator of vitamin B‐12 status. Am. J. Clin. Nutr. 103: 250–257. 26607937 10.3945/ajcn.115.116509

[65] Yajnik, C.S. , S.S. Deshpande , A.A. Jackson , *et al* 2008 Vitamin B12 and folate concentrations during pregnancy and insulin resistance in the offspring: the Pune Maternal Nutrition Study. Diabetologia 51: 29–38. 17851649 10.1007/s00125-007-0793-y PMC2100429

[66] Stewart, C.P. , P. Christian , K.J. Schulze , *et al* 2009 Antenatal micronutrient supplementation reduces metabolic syndrome in 6‐ to 8‐year‐old children in rural Nepal. J. Nutr. 139: 1575–1581. 19549749 10.3945/jn.109.106666

[67] Cooney, C.A. , A.A. Dave & G.L. Wolff . 2002 Maternal methyl supplements in mice affect epigenetic variation and DNA methylation of offspring. J. Nutr. 132(8 Suppl.): 2393S–2400S. 12163699 10.1093/jn/132.8.2393S

[68] Dolinoy, D.C. , J.R. Weidman , R.A. Waterland & R.L. Jirtle . 2006 Maternal genistein alters coat color and protects Avy mouse offspring from obesity by modifying the fetal epigenome. Environ. Health Perspect. 114: 567–572. 16581547 10.1289/ehp.8700 PMC1440782

[69] Morgan, H.D. , H.G.E. Sutherland , D.I.K. Martin & E. Whitelaw . 1999 Epigenetic inheritance at the agouti locus in the mouse. Nat. Genet. 23: 314–318. 10545949 10.1038/15490

[70] Waterland, R.A. & R.L. Jirtle . 2003 Transposable elements: targets for early nutritional effects on epigenetic gene regulation. Mol. Cell. Biol. 23: 5293–5300. 12861015 10.1128/MCB.23.15.5293-5300.2003 PMC165709

[71] Modabbernia, A. , E. Velthorst & A. Reichenberg . 2017 Environmental risk factors for autism: an evidence‐based review of systematic reviews and meta‐analyses. Mol. Autism 8: 13. 28331572 10.1186/s13229-017-0121-4 PMC5356236

[72] Gao, Y. , C. Sheng , R.‐H. Xie , *et al* 2016 New perspective on impact of folic acid supplementation during pregnancy on neurodevelopment/autism in the offspring children—a systematic review. PLoS One 11: e0165626. 27875541 10.1371/journal.pone.0165626 PMC5119728

[73] Zhao, M. , X. Wang , M. He , *et al* 2017 Homocysteine and stroke risk: modifying effect of methylenetetrahydrofolate reductase C677T polymorphism and folic acid intervention. Stroke. 48:1183–1190. 28360116 10.1161/STROKEAHA.116.015324

[74] Xu, A. , X. Cao , Y. Lu , *et al* 2016 A meta‐analysis of the relationship between maternal folic acid supplementation and the risk of congenital heart defects. Int. Heart J. 57: 725–728. 27829639 10.1536/ihj.16-054

[75] Qin, X. , J. Li , J.D. Spence , *et al* 2016 Folic acid therapy reduces the first stroke risk associated with hypercholesterolemia among hypertensive patients. Stroke 47: 2805–2812. 27729579 10.1161/STROKEAHA.116.014578

[76] Liu, S. , K.S. Joseph , W. Luo , *et al* 2016 Effect of folic acid food fortification in Canada on congenital heart disease subtypes. Circulation 134: 647–655. 27572879 10.1161/CIRCULATIONAHA.116.022126 PMC4998126

